# Extracellular volume assessed by CMR T1-mapping correlates with histologically determined amount of diffuse fibrosis in DCM

**DOI:** 10.1186/1532-429X-16-S1-M8

**Published:** 2014-01-16

**Authors:** Fabian aus dem Siepen, Sebastian Buss, Florian Andre, Marius Keller, Sebastian A Seitz, Grigorios Korosoglou, Evangelos Giannitsis, Hugo A Katus, Henning Steen

**Affiliations:** 1 Department of Cardiology, University of Heidelberg, Heidelberg, Germany

## Background

Diffuse myocardial fibrosis (MF) in dilated cardiomyopathy (DCM) is closely related to systolic and diastolic cardiac failure and could be identified as a major independent value prediciting clinical outcome. It results from elevated levels of collagen and is therefore associated with an expansion of the extracellular volume (ECV). Up to now, the detection of MF requires myocardial biopsy. Recent reports indicate that CMR T1-Mapping has the potential to detect MF non-invasively. We compared the ECV values derived from T1-mapping with the collagen volume fraction (CVF) as meausured in histological samples.

## Methods

**Figure 1:**
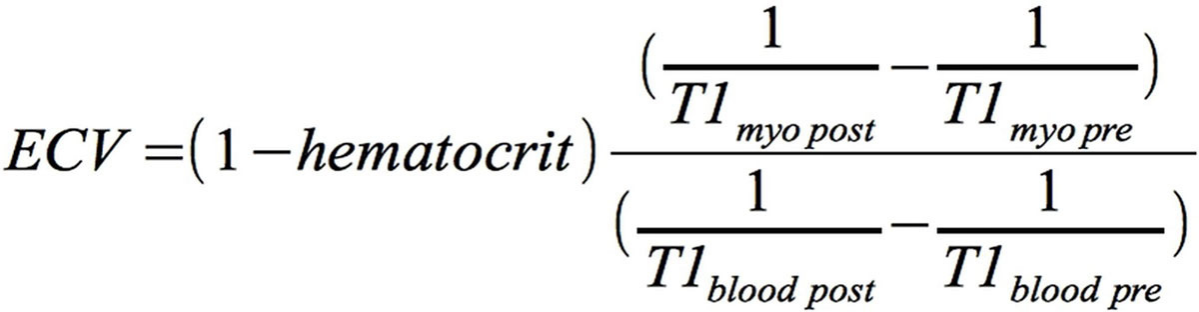
**T1 myo pre: native T1 relaxation time for myocardium T1 myo post: post-contrast T1 relaxation time for myocardium T1 blood pre: native T1 relaxation time for blood T1 blood post: post-contrast T1 relaxation time for blood**.

All CMR examinations were performed in a 1.5 T CMR scanner (Achieva, Philips Healthcare). Short axis slices covering the left ventricle were acquired using SSFP-sequences to measure volumes and ejection fraction. T1- relaxation times were measured from 24 patients (54 ± 13 years,16 males) with DCM before and 15 minutes after injection of gadolinium-DTPA contrast agent (Magnevist, 0.2 mmol/kg body weight). T1-maps were created out of 11 mid-ventricular short axis views with increasing inversion times (TI; 100-4400 msec.) using a single breath-hold modified Look-Locker inversion-recovery sequence (MOLLI, TR/TE = 3.5/1.8 msec, flip angle = 35°) in late diastole. The formula for calculating the extracellular volume fractions is given in Figure 1. Myocardial biopsies were taken and stained with Acid Fuchsin Orange-G (AFOG). Tissue collagen content was quantified histologically by using an automated image analysis system and correlated with ECV measurements.

## Results

**Figure 2:**
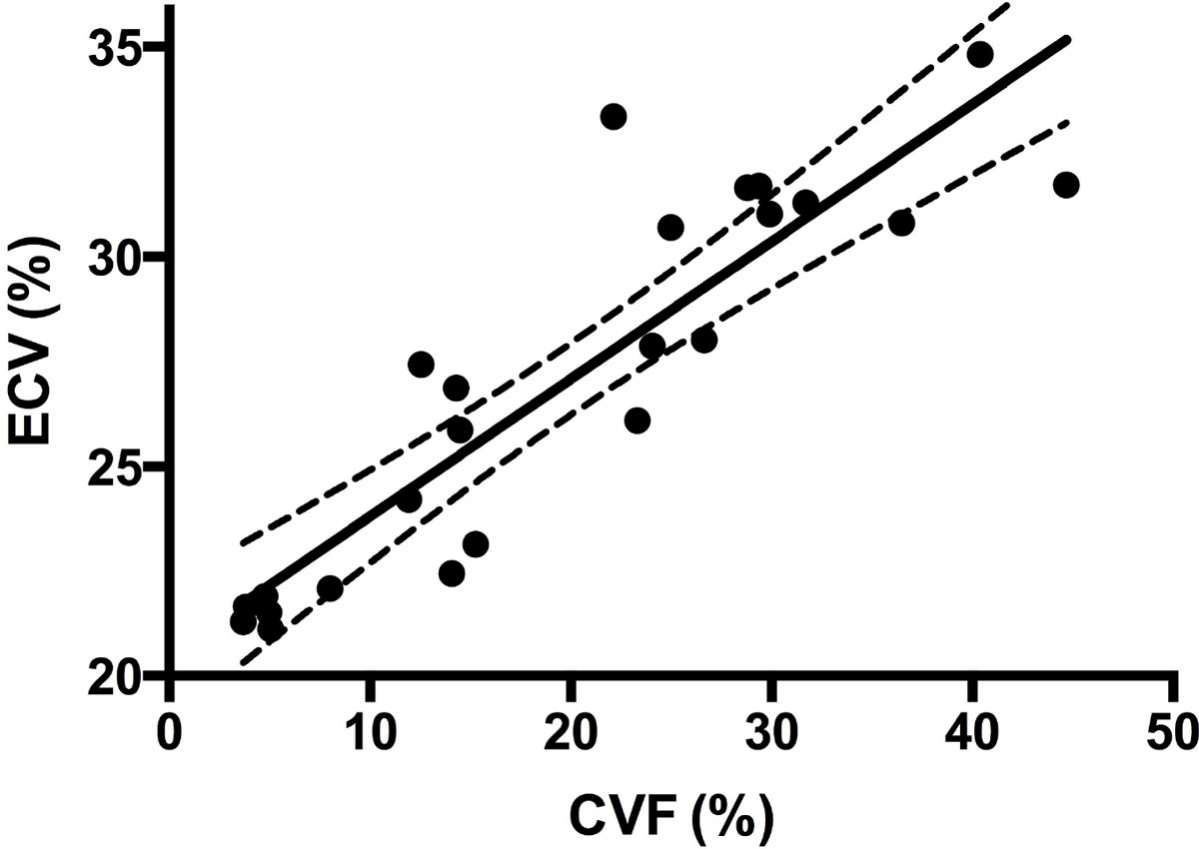
**ECV: extracellular volume CVF: collagen volume fraction**.

The DCM patients had a mean left ventricular ejection fraction (LVEF) of 39 ± 16%. Average ECV fraction was 27 ± 4% and CVF was 19 ± 4% in our study population. There was a strong correlation between ECV and CVF (r = 0.85; p = 0.01, Figure 2). Patients with severe reduced cardiac function (LVEF < 30%) had the highest CVF and ECV values.

## Conclusions

CMR-based ECV assessment may offer the potential to serve as a non-invasive tool for the quantification of MF in DCM and possibly in other cardiomyopathies as well. Further studies are needed to investigate its impact on clinical outcome prediction in DCM patients.

## Funding

None.

